# Pre-pregnancy care in general practice in England: cross-sectional observational study using administrative routine health data

**DOI:** 10.1186/s12889-025-21728-1

**Published:** 2025-03-22

**Authors:** Yangmei Li, Jennifer J. Kurinczuk, Fiona Alderdice, Maria A. Quigley, Oliver Rivero-Arias, Julia Sanders, Sara Kenyon, Dimitrios Siassakos, Nikesh Parekh, Suresha De Almeida, Claire Carson

**Affiliations:** 1 NIHR Policy Research Unit in Maternal and Neonatal Health and Care, National Perinatal Epidemiology Unit, Nuffield Department of Population Health, University of Oxford, Old Road Campus, Headington, Oxford, OX3 7LF UK; 2 School of Healthcare Sciences, Cardiff University, Cardiff, UK; 3 Institute of Applied Health Research, University of Birmingham, Birmingham, UK; 4 EGA Institute for Women’s Health, University College London, London, UK; 5 Wellcome/EPSRC Centre for Interventional and Surgical Sciences (WEISS), London, UK; 6 Public Health and Wellbeing, Royal Borough of Greenwich, London, UK; 7 NHS South West London CCG, London, UK

**Keywords:** Preconception care, General practice, General practitioners, Primary health care, Health promotion

## Abstract

**Background:**

Optimising women's pre-pregnancy health is a policy priority for benefits spanning pregnancy and throughout the mother and baby's life. In the UK pre-pregnancy care (PPC) tends to be delivered in primary care, with the onus on women to seek services. We aimed to describe women’s engagement with General Practice (GP) in the year preceding pregnancy, including specific PPC; to explore whether women with recognised risk factors for poor pregnancy outcomes receive targeted care.

**Methods:**

Data for women aged 18-48yrs and registered for $\ge$ 12 months with a GP on 01/01/2017, were drawn from English Clinical Practice Research Datalink (CPRD) GOLD, a source of electronic health record data. Demographic characteristics, lifestyle factors and health conditions were described. CPRD Pregnancy Register and linked hospital data were used to identify pregnancies in 2017/18 and to describe PPC in the year preceding pregnancy.

**Results:**

Of 193,578 women included, 14,326 had a confirmed pregnancy. 7.6% of the pregnant women had records indicating specific PPC in the preceding year, whilst 41.0% had records of health promotion (advice on nutrition, smoking, weight, alcohol and contraception). More women with pre-existing medical conditions received health promotion (46.0%-83.9% for various risk groups), although the levels of PPC remained low (4.7%-14.9%).

**Conclusions:**

PPC was rarely recorded, likely reflecting low levels of consultation for, or discussion of, pregnancy planning. This represents a missed opportunity for maximising women’s health, particularly in those with recognised risk factors for poor pregnancy, perinatal and longer-term outcomes.

**Supplementary Information:**

The online version contains supplementary material available at 10.1186/s12889-025-21728-1.

## Background

Engaging women in improving their health prior to pregnancy has been identified as an important public health policy [1, 2]. Optimising a woman’s health before she conceives benefits her at the time, in her subsequent pregnancy and birth, and has positive effects for her longer-term health and that of her infant [3, 4]. However, interventions cannot solely target those planning to conceive; just 55% of UK births follow a “planned ‘’ pregnancy [1, 5]. Thus women of reproductive age are an important target population for public health interventions designed to have lasting impacts on health and, as a consequence, improve health outcomes, reduce health care costs and demands on services.

Women with pre-existing conditions are more likely to experience adverse pregnancy, maternal and perinatal health outcomes compared with those without [6–11]. For example, pre-existing hypertension, diabetes and obesity are all risk factors for pre-eclampsia [6, 7], and contribute to an elevated risk of preterm birth [8, 9]. Women with pre-existing health conditions account for a non-negligible proportion of women of reproductive age and proactive management of the conditions prior to pregnancy can improve their pregnancy wellbeing and outcomes [9, 12–16].

Current guidance in England and other European countries states that pre-pregnancy care (PPC) should encompass a wide range of checks and advice. This includes health improvements such as stopping smoking, eating a healthy diet, maintaining a healthy weight, and being physically active, as well as addressing mental health and psychosocial stress [17, 18]. Pregnancy-specific advice entails taking folic acid supplements, and discussing birth spacing with women who already have a child. Specific high-risk groups are also highlighted, for example where women have pre-existing health conditions that must be managed through pregnancy (e.g. diabetes or epilepsy) or take medications detrimental to the foetus (e.g. valproate).

In the Women’s Health Strategy for England, the Department of Health and Social Care has stated their 10-year ambition to “support women and their partners to optimise their health and wellbeing prior to conception” [19]. Although guidance exists, a specific ‘pre-pregnancy’ General Practice (GP) appointment is not currently standard care in England and the National Health Service (NHS) recommends women seek advice only if they are in specific risk groups. It is therefore unclear how many women receive any pre-conception advice prior to becoming pregnant (or between pregnancies), or whether GPs discuss these issues unless the women themselves start the conversation.

The overall aim of this study is to gain a better understanding of women’s engagement with GP services in the year prior to pregnancy, and specifically PPC received in GP services. Specific objectives include:

i) To establish whether it is feasible to identify PPC using routine health records;
ii) To investigate whether women who have pre-existing risk factors are targeted for PPC;
iii) To describe women’s overall engagement with GP services in the year prior to pregnancy to explore opportunities to optimise women’s health before pregnancy.

## Methods

### The data sources

This study was approved by the CPRD Independent Scientific Advisory Committee (ISAC, protocol number: 20_000220). Primary care data were drawn from the Clinical Practice Research Datalink (CPRD) GOLD dataset, which covers around 6.9% of the UK population, and is broadly representative in terms of age, sex and ethnicity [20]. Pregnancy data were available in the CPRD Pregnancy Register (PR) and CPRD Mother Baby Link (MBL), and in the maternity tables from linked Hospital Episode Statistics (HES) Admitted Patient Care records [21]. CPRD PR uses an algorithmic approach to identify pregnancies in the coded primary care records [20, 22]. CPRD MBL identifies births in women’s records and links mothers with babies in the same family within the appropriate time-period. HES maternity data contains details of hospital and out of hospital births where care is provided by NHS staff, including some details about the baby [21, 23]. Linkage to HES records allows pregnancies to be included that may have been missed in the primary care records.

### Study population and setting

All women with research-quality data, registered at an English GP participating in CPRD were included if they were aged 18-48yrs on 01/01/2017, had a year of prior registration and were eligible for hospital data linkage. The required registration period ensured that they would be eligible for inclusion in the Pregnancy Register and that there would be sufficient time for their records to reflect pre-conception care, health and demographic characteristics.

### Identification of pregnancies

We used the PR, augmented by the MBL and HES maternity data, to identify those women who were not pregnant on 31st December 2016 but subsequently became pregnant by 31st December 2018. An algorithm was developed to amalgamate the three data sources [24]. Overlapping pregnancy records were grouped together and reduced to one record per pregnancy. Additional cleaning removed records that were likely to be non-contemporaneous or partial records erroneously identified as a separate unique pregnancy by the original PR algorithm.

### Deriving variables

Records from the CPRD clinical, referral, therapy and test files and linked indices of multiple deprivation (IMD) data were used to derive sociodemographic and health-related variables, using code lists (see Table 1 and Additional file 1 for details of variables and categorisation). Code lists were adapted from previously published lists, using the CPRD GOLD code browser (version 3.0.0) and clinical input (Additional file 1). The selected pre-existing health conditions are recognised risk factors for poor pregnancy, perinatal and longer-term outcomes, particularly if severe or poorly managed. Women’s interaction with the GP services, including face-to-face contact with general practitioners and/or nurses and cervical screening appointments were derived to explore opportunities for GPs to optimise women’s health before pregnancy.

**Table 1:** Characteristics of study population, and subset of women who became pregnant during follow up

	All women of reproductive age^a^	Women who became pregnant^b^
	**Number (%)**	**Number (%)**
	N = 193,578	N = 14,326
**Sociodemographic characteristics**		
**Age, mean (SD)**	34.4 (8.8)	30.5 (5.8)
< 20 yrs	9307 (4.8)	324 (2.3)
20–24 yrs	23,910 (12.4)	2100 (14.7)
25–29 yrs	28,988 (15.0)	3733 (26.1)
30–34 yrs	31,435 (16.2)	4482 (31.3)
35–39 yrs	33,722 (17.4)	2808 (19.6)
40–44 yrs	35,067 (18.1)	775 (5.4)
≥ 45 yrs	31,149 (16.1)	104 (0.7)
**Ethnic group**		
White British	91,629 (68.8)	7235 (69.3)
White other	18,968 (14.3)	1372 (13.1)
Mixed	2227 (1.7)	187 (1.8)
Asian or Asian british	10,062 (7.6)	877 (8.4)
Black or Black british	6383 (4.8)	514 (4.9)
Chinese or other	3847 (2.9)	255 (2.4)
*Missing*	*60,462 (31.2)*	*3886 (27.1)*
**Practice IMD quintiles**		
1 (least deprived)	33,966 (17.6)	2367 (16.5)
2	36,158 (18.7)	2418 (16.9)
3	37,700 (19.5)	2776 (19.4)
4	30,445 (15.7)	2515 (17.6)
5	55,309 (28.6)	4250 (29.7)
**Health status and risk behaviours**		
**BMI (kg/m**^**2**^**), mean (SD)**	26.4 (6.6)	26.0 (6.2)
< 18.5	7,087 (4.1)	555 (4.3)
18.5–24.9	80,015 (46.6)	6329 (48.9)
25–29.9	44,171 (25.7)	3285 (25.4)
≥ 30	40,436 (23.6)	2776 (21.4)
*Missing*	*21,869 (11.3)*	*1381 (9.6)*
** Smoking**		
Never smokers	104,803 (55.8)	7446 (52.8)
Former smokers	47,762 (25.4)	3608 (25.6)
Current smokers	35,410 (18.8)	3062 (21.7)
*Missing*	*5603 (2.9)*	*210 (1.5)*
**Current health conditions**		
Diabetes mellitus	3088 (1.6)	130 (0.9)
Hypertension	5509 (2.9)	132 (0.9)
Asthma (ever diagnosed)	33,089 (17.1)	2570 (17.9)
Actively managed asthma (ever diagnosed + treated in the last year)	13,883 (7.2)	908 (6.3)
Epilepsy (ever diagnosed)	2850 (1.5)	195 (1.4)
Actively managed epilepsy (ever diagnosed + treated in the last year)	1422 (0.7)	87 (0.6)
Cardiovascular disease (CVD)	667 (0.3)	18 (0.1)
Common mental disorders (depression and/or anxiety, ever diagnosed)	65,647 (33.9)	4629 (32.3)
Common mental disorders (depression and/or anxiety, recent)	29,251 (15.1)	2097 (14.6)
Polycystic ovary syndrome (PCOS)	9224 (4.8)	837 (5.8)

^a^Study population consists of all women with research quality data who were registered at an English GP and were aged 18–48 on 01/01/2017, had a year of prior registration and were consent to hospital data linkage. Characteristics and conditions are shown as of 01 Jan 2017

^b^Study population consists of women eligible for the study who had a pregnancy which started in 2017 or 2018. Characteristics and conditions are shown as at the start of the index pregnancy

16,453 pregnancies started between 2017–2018, for women who had more than one pregnancy during this period, only the first was kept (n = 14,326, 87.1% of all pregnancies starting in 2017–2018)

Proportions are presented as the % of non-missing, unless in italics, where % is of all women

### Identification of pre-pregnancy care

To capture PPC, records were restricted to the year before the baseline date (01/01/2017), for all eligible women of reproductive age (to include those who did not go on to conceive), and the year prior to pregnancy for those who became pregnant, respectively. Diagnostic and symptom codes relating to PPC were extracted from the women’s clinical and referral records in CPRD using pre-specified code lists (Additional file 2) to explore the feasibility of identifying women receiving PPC. Two types of care were considered: specific PPC (folic acid advice, fertility discussions, general PPC and advice) and general health promotion (nutrition, smoking cessation, weight management, alcohol advice, contraception).

### Analysis

Summary statistics were used to describe sociodemographic characteristics, health behaviours and pre-existing conditions among the eligible women, and among those with a pregnancy recorded in 2017- 2018. We then described the PPC women received, for all women, all pregnant women and in those with recognised risk factors for poor pregnancy, perinatal and longer-term outcomes. For women with more than one pregnancy in the study period, the first pregnancy was included. Additionally, we explored PPC by women’s characteristics among those with a pregnancy recorded in 2017- 2018.

To explore opportunities for GPs to optimise women’s health before pregnancy starts, we calculated the percentage of women who had any engagement with the GP service in the year prior to pregnancy for all women with a pregnancy in 2017–2018, and for those with pre-existing conditions. We additionally described any evidence of active management of their pre-existing condition (Additional file 3).

All analyses were performed with Stata 17 [25].

## Results

### Characteristics of women

The characteristics of women of reproductive age and the prevalence of pre-existing health conditions are shown in Table 1. The mean age (SD) of the 193,578 women of reproductive age included in our analysis was 34.4 years (8.8). White British was the most frequently recorded ethnicity (68.8%), 14.3% were recorded as White other, and 16.9% were from other ethnic groups. More than a quarter (28.6%) of women were registered with practices in the most deprived IMD quintile.

Almost half of the women of reproductive age were overweight or obese (Table 1). More than half had never smoked (55.8%), 25.4% were former smokers and 18.8% current smokers. The prevalence of ever diagnosed asthma was 17.1% and common mental disorders was 33.9%. Records indicated that 4.8% of women had a diagnosis of PCOS, 2.9% of women were living with hypertension and 1.6% with diabetes. Between 2017 and 2018, 14,326 women had a recorded pregnancy (Table 1). The pregnant women were, on average, younger and healthier than the general population of women of reproductive age. However, of the women who became pregnant, about two thirds (n = 9,528, 66.5%) still had had at least one pre-existing health condition placing them at higher risk of poorer outcomes.

### Pre-pregnancy care

PPC was rarely recorded; only 2,640 women had specific PPC codes (1.4% of women of reproductive age, Table 2). Of the women with PPC records, less than half had evidence of a pregnancy in the following 24 months (n = 1,093, Table 2). On average, women aged 30–44 years, Asian or Asian British women, nulliparous women and those from less deprived areas were more likely to have records of PPC (Additional file 4, Supplementary Table).

**Table 2:** Provision of pre-pregnancy care in all women, women who became pregnant in 2017–2018, and higher risk women who became pregnant in 2017–2018

			**Among women who became pregnant**								
**Type of care or advice**	**All women**^**a**^	**Women who became pregnant in 2017-2018**^**b**^	**Current smokers**	**Women overweight and obese**	**Women with mental health issues**	**Women with diabetes**	**Women with hypertension**	**Women with asthma**	**Women with actively managed asthma**^**c**^	**Women with epilepsy**	**Women with actively managed epilepsy**^**c**^
	**Number (%)**	**Number (%)**	**Number (%)**	**Number (%)**	**Number (%)**	**Number (%)**	**Number (%)**	**Number (%)**	**Number (%)**	**Number (%)**	**Number (%)**
	N = 193,578 (100.0)	N = 14,326 (100.0)	N = 3062 (100.0)	N = 6061 (100.0)	N = 4629 (100.0)	N = 130 (100.0)	N = 132 (100.0)	N = 2570 (100.0)	N = 908 (100.0)	N = 195 (100.0)	N = 87 (100.0)
**Specific pre-pregnancy care and advice**	2640 (1.4)	1093 (7.6)	143 (4.7)	472 (7.8)	318 (6.9)	14 (10.8)	10 (7.6)	206 (8.0)	85 (9.4)	18 (9.2)	13 (14.9)
** Folic acid advice**	130 (0.1)	25 (0.2)	≤ 5	8 (0.1)	12 (0.3)	≤ 5	≤ 5	≤ 5	≤ 5	≤ 5	≤ 5
** Fertility concerns**	1609 (0.8)	721 (5.0)	86 (2.8)	309 (5.1)	202 (4.4)	≤ 5	7 (5.3)	132 (5.1)	55 (6.1)	12 (6.2)	7 (8.1)
** General pre-pregnancy care and advice**	809 (0.4)	307 (2.1)	46 (1.5)	141 (2.3)	100 (2.2)	8 (6.2)	≤ 5	60 (2.3)	25 (2.8)	6 (3.1)	6 (6.9)
** Referral for specific pre-pregnancy care and advice**	685 (0.4)	323 (2.3)	38 (1.2)	140 (2.3)	69 (1.5)	≤ 5	≤ 5	60 (2.3)	21 (2.3)	≤ 5	≤ 5
**General health promotion**	80,293 (41.5)	5869 (41.0)	1998 (65.3)	2862 (47.2)	2249 (48.6)	109 (83.9)	72 (54.6)	1302 (50.7)	525 (57.8)	90 (46.2)	40 (46.0)
** Nutrition**	4033 (2.1)	212 (1.5)	45 (1.5)	109 (1.8)	78 (1.7)	20 (15.4)	11 (8.3)	54 (2.1)	29 (3.2)	≤ 5	≤ 5
** Smoking cessation**	21,313 (11.0)	1816 (12.7)	1623 (53.0)	816 (13.5)	891 (19.3)	36 (27.7)	21 (15.9)	467 (18.2)	200 (22.0)	30 (15.4)	15 (17.2)
** Weight management**	55,462 (28.7)	3842 (26.8)	900 (29.4)	2073 (34.2)	1382 (29.9)	98 (75.4)	62 (47.0)	842 (32.8)	358 (39.4)	54 (27.7)	25 (28.7)
** Alcohol drinking advice**	3497 (1.8)	158 (1.1)	49 (1.6)	72 (1.2)	64 (1.4)	6 (4.6)	6 (4.6)	40 (1.6)	21 (2.3)	≤ 5	≤ 5
** Family planning and contraception**	33,870 (17.5)	2453 (17.1)	617 (20.2)	1097 (18.1)	872 (18.8)	17 (13.1)	17 (12.9)	533 (20.7)	167 (18.4)	36 (18.5)	14 (16.1)
** Referral for general health promotion**	546 (0.3)	40 (0.3)	14 (0.5)	35 (0.6)	20 (0.4)	≤ 5	≤ 5	9 (0.4)	≤ 5	≤ 5	≤ 5

^a^Study population consists of all women with research quality data who were registered at an English GP and were aged 18–48 on 01/01/2017, had a year of prior registration and were consent to hospital data linkage. Pre-pregnancy care shown in the column is related to care received in 2016

^b^Study population consists of women eligible for the study who had a pregnancy starting in 2017 or 2018. Pre-pregnancy care shown in the column is related to care received in the year prior to the start of the index pregnancy

16,453 pregnancies started between 2017–2018, for women who had more than one pregnancy during this period, only the first was kept (n = 14,326, 87.1% of all pregnancies starting in 2017–2018)

^c^Ever diagnosed and treated in the last year

For cells where there are no more than 5 cases, details have been removed

Of the 14,326 women who subsequently became pregnant in the study period, 7.6% had PPC recorded in the preceding 12 months, and the prevalence remained low in the sub-groups of women with pre-existing conditions (4.7% to 14.9%, Table 2).

Around 41.5% of all women of reproductive age and a similar proportion (41.0%) of those who became pregnant had general health promotion records in the previous year (Table 2). Overall, more women with pre-existing conditions received general health promotion (between 46.0% and 83.9% for various risk groups) compared with all pregnant women.

### Opportunities for PPC or discussion of pregnancy intentions

Almost all pregnant women had contact with GP services in the year prior to the start of pregnancy (96.9%, Table 3). Just under four fifths (79.7%) had one or more face-to-face appointments with a general practitioner, and 44.2% with a nurse. About one fifth had records for cervical screening appointments in the year prior to pregnancy. The proportions of women with pre-existing conditions who engaged with GP services in the year prior to the pregnancy were generally higher (between 97.7% and 100.0%, Table 3). Evidence of active management of a pre-existing condition in the year prior to pregnancy varied by diagnosis (Table 3).

**Table 3:** Opportunities for pre-pregnancy care in women who became pregnant in 2017–2018, and higher risk women who became pregnant in 2017–2018

		**Among women who became pregnant**								
	**Women who became pregnant in 2017-2018**^**a**^	**Current smokers**	**Women overweight and obese**	**Women with mental health issues**	**Women with diabetes**	**Women with hypertension**	**Women with asthma**	**Women with actively managed asthma**^**b**^	**Women with epilepsy**	**Women with actively managed epilepsy**^**b**^
	**N = 14,326**	N = 3062	N = 6061	N = 4629	N = 130	N = 132	N = 2570	N = 908	N = 195	N = 87
	**%**	%	%	%	%	%	%	%	%	%
**Contact with GP services**										
**Face-to-face contact with a general practitioner (%)**	**79.7**	81.5	82.1	88.1	92.3	83.3	83.6	89.8	83.6	92.0
**Face-to-face contact with a nurse (%)**	**44.2**	44.2	46.6	48.8	64.6	47.7	53.2	61.1	45.1	39.1
**Face-to-face contact with a general practitioner or a nurse (%)**	**86.6**	86.9	88.5	92.2	95.4	89.4	90.7	95.4	88.2	94.3
**Any contact with GP surgery including letter or phone call (%)**	**96.9**	98.2	97.8	98.6	99.2	97.7	98.3	99.6	98.0	100.0
**Cervical screening appointments**	**21.6**	17.1	21.8	22.5	20.0	19.7	21.8	22.6	18.0	17.2
**Active management of the condition**	**N/A**	**Entity type codes**^**c**^	**Entity type codes**^**c**^	**N/A**	**Blood sugar monitoring and Hb1Ac**	**Blood pressure check**	**Asthma review**	**Asthma review**	**Epilepsy review**	**Epilepsy review**
		48.3	34.4		68.5	80.3	21.4	51.9	5.6	10.3

^a^Study population consists of women eligible for the study who had a pregnancy starting in 2017 or 2018. Care shown in the column is related to care received in the year prior to the start of the index pregnancy

16,453 pregnancies started between 2017–2018, for women who had more than one pregnancy during this period, only the first was kept (n = 14,326, 87.1% of all pregnancies starting in 2017–2018)

^b^Ever diagnosed and treated in the last year

^c^Entity type codes are additional data entered under the structured administrative data area in the GP software

## Discussion

### Summary

PPC is rarely recorded in coded electronic health record (EHR) data. Among pregnant women, only 7.6% had records of pre-pregnancy care, including folic acid advice, in the year prior to pregnancy; 41.0% had general health promotion records. While women with pre-existing conditions generally received higher levels of general health promotion than the overall population of pregnant women, the levels of PPC were similar or only slightly higher on average. Despite the low prevalence of recorded PPC, most of women interacted with their GP services in the year prior to the start of pregnancy, presenting opportunities for GPs to support women to improve their health and wellbeing before pregnancy.

### Strengths and limitations

CPRD GOLD is broadly representative of England’s population, identifies pregnancy and those at higher risk, and therefore allows us to describe consultations before pregnancy, and explore PPC in those with pre-existing conditions. Augmentation of primary care data using hospital maternity records allows the identification of pregnancies that were omitted in GP coded data. As such, we can describe PPC in women who subsequently have a pregnancy. However, pregnancies ending in termination or miscarriage may still be underestimated as GPs are not be informed in all cases. As these pregnancies may be excluded from the denominator, this may overestimate PPC provision among those who became pregnant. Data on pregnancy intention was not available, and therefore we cannot specifically explore the provision of PPC within those hoping to conceive.

The use of detailed code lists, drawing on diagnosis, symptoms, prescriptions and referrals maximises the opportunity to identify the variables of interest in EHRs. However, data quality is dependent on recording by GPs. For example, free-text notes regarding PPC would be missed, and some non-specific codes such as ‘had a chat to patient’ may encompass PPC but would not have been recognised as such. Women with the pre-existing conditions that place them at higher risk of poorer outcomes, particularly epilepsy, may be managed in a hospital setting. The GP EHRs may not reflect these, as hospital letters are not always coded and available for quantitative research. Using coded GP data may therefore underestimate the provision of some aspects of care.

### Comparison with existing literature

Despite the growing recognition of PPC as a crucial component of maternal health care [26], research on its current provision or uptake is limited and often only conducted in higher risk population [27]. Research often focuses on antenatal interventions, with fewer studies shedding light on the proactive health measures taken by women before pregnancy [28, 29]. There is also a lack of available effective pre-pregnancy interventions, for example, for weight loss in the pre-pregnancy period [30]. This evidence gap poses a challenge to policymakers and healthcare providers striving to implement effective strategies for PPC.

Historically, PPC has focused on women actively planning a pregnancy [1]. Yet, given that nearly half of all pregnancies are not planned [5], timely delivery of PPC presents a considerable challenge. Our finding adds to the evidence that a substantial proportion of women in their childbearing years only seek care after becoming pregnant [1], emphasising the need to broaden the scope and timing of preconception health initiatives and the importance of a focus on improvements in the public health of the population in general.

### Implications for research and/or practice

The responsibility for PPC currently falls largely on women, who are expected to seek appropriate medical guidance [31]. However, our study indicates that the majority of women do not engage in PPC via primary care services in the year prior to pregnancy, despite having contact with their GP for other health issues during the same period. Those who do may be either proactive planners or women who have faced challenges in conceiving and are now seeking fertility assistance [32]. This suggests that although primary care represents a highly accessible channel for reaching women, both healthy and at higher risk, it is not being fully utilised for delivering PPC. While guidelines for healthcare professionals on delivering PPC do exist [17, 18, 33], there is a need for greater clarity regarding the responsibilities and the most effective methods for delivering PPC. Specifically, it is crucial to define the roles of GPs and health services in promoting PPC. This process should engage all stakeholders, including GPs, women, and other health services.

Identifying and addressing barriers to PPC is a crucial step in improving PPC, both from the perspective of women seeking care and GPs providing it. Known challenges for GPs include inadequate training and proficiency in delivering health guidance [34], time constraints within consultations, the low prevalence of women proactively seeking preconception care, multiple competing preventive care priorities in general practice, concerns regarding the affordability and accessibility of preconception care, and insufficient resources for supporting the implementation of preconception care guidelines [35, 36]. Barriers for women include, but are not limited to, a lack of understanding regarding the importance and benefits for planning a pregnancy [32, 37], quicker-than-expected conception, logistical challenges in attendance [38], unsupportive staff and overly busy clinics [37]. Although PPC has shown effectiveness for women with serious health conditions, its broader impact on improving pregnancy outcomes appears limited [39], which may affect the motivation of both GPs and women to engage. Furthermore, the COVID-19 pandemic generally led to reduced access to healthcare services, which may have disproportionately affected certain population groups who were already less likely to use primary care before the pandemic [40, 41].

Addressing these barriers requires a multifaceted approach, including enhanced GP training, better resource allocation, public education campaigns to raise awareness, and systemic changes to improve accessibility and integration of preconception care into routine general practice [36]. Further research is warranted to assess the wider benefits of PPC and to develop evidence-based strategies for overcoming these challenges.

While considerable efforts are made to prevent unwanted pregnancy, there is a notable lack of public health emphasis on considering the optimal timing for conception and improving overall health for pregnancy. By integrating PPC into general health promotion initiatives for women's health, we can ensure that women, whether or not they are actively planning, are informed and empowered to make healthier choices [32].

High-risk groups, particularly those with pre-existing health conditions, represent an easily identifiable demographic for PPC. On the other hand, women in the highest risk groups may choose termination of pregnancy if they have not received appropriate PPC. However, our results suggest that even within this group, there is insufficient awareness and engagement. Research indicates that high-risk women, their healthcare professionals, and the healthcare system may not prioritise planning for pregnancy [42], and women may not be aware of the need to do so [27]. Coordinating care between primary and consultant-led services poses additional challenges, necessitating clear guidelines on communication, responsibility and jurisdiction. Addressing these challenges is essential to ensuring that those at increased risk receive the most beneficial PPC.

General practices could employ various methods to promote PPC, including providing information on practice websites, signposting via text messages or social media, and distributing physical posters and leaflets within the surgery. However, these messages will not reach all women and are more likely to be noticed by women actively planning to conceive or who have a higher level of health awareness. A conversation with a trained health care professional, particularly for women who have existing health concerns or risk factors, is more likely to ensure that women engage with the information provided. Improving the coding of PPC within EHRs, particularly by including detailed information about discussions between healthcare providers and patients, as well as the information or guidance given to women, is imperative, as monitoring and evaluating the effectiveness of PPC interventions rely on accurate data coding.

While the findings of this study are most relevant to England and may have limited generalisability to other countries and healthcare settings, they offer insights with global relevance. In countries with similar health systems, clarifying roles and improving the coding and monitoring of routine health data [29] could contribute to enhancing PPC. For countries with weaker health infrastructures, prioritising the identification of barriers and reallocating resources to address them might take precedence. Regardless of the healthcare context, raising awareness about the importance and advantages for planning a pregnancy could play a key role in reducing unplanned pregnancies and improving preconception health. Moreover, integrating PPC into broader health promotion strategies in an equitable way could help address missed opportunities across all healthcare systems.

## Conclusions

Pre-pregnancy care is rarely recorded in primary care in England, likely reflecting low levels of consultations for pregnancy planning, even among those with pre-existing recognised health conditions that place them at higher risk for poorer pregnancy and health outcomes. This represents a missed opportunity for promoting pre-pregnancy and subsequent health. The current state of pre-pregnancy care calls for a shift towards a more proactive and collaborative approach to meet the policy ambition of the Women’s Health Strategy. It is necessary to clarify responsibilities, address barriers, and integrate appropriate pre-pregnancy care into broader health promotion initiatives, alongside enhanced coding of routine health data and monitoring.

## Supplementary Information

Additional file 1. Derivation of variables.

Additional file 2. Identification of pre-pregnancy care and advice.

Additional file 3. Code lists for active management of medical conditions.

Additional file 4. Supplementary Table Provision of pre-pregnancy care by characteristics of women in those who became pregnant in 2017–2018.

## Data Availability

This study is based on data from the Clinical Practice Research Datalink obtained under licence from the UK Medicines and Healthcare products Regulatory Agency. The data is provided by patients and collected by the NHS as part of their care and support. The interpretation and conclusions contained in this study are those of the authors alone. Copyright © 2023, re-used with the permission of The Health & Social Care Information Centre. All rights reserved. The datasets generated and/or analysed during the current study are not publicly available, as the data were provided by the Clinical Practice Research Datalink under a contractual agreement that does not permit the sharing of data. Study documentation is available on request from the corresponding author.

## Abbreviations

CPRD: Clinical Practice Research Datalink
EHR: Electronic health record
GP: General practice
HES: Hospital Episode Statistics
IMD: Index of Multiple Deprivation
ISAC: Independent Scientific Advisory Committee
MBL: Mother Baby Link
NHS: National Health Service
PR: Pregnancy Register
PPC: Pre-pregnancy care
SD: Standard deviation
