# Supplementary material for: Pre-pregnancy care in general practice in England: cross-sectional observational study using administrative routine health data
Source: BMC Public Health. 2025 Mar 22;25:1101. doi: 10.1186/s12889-025-21728-1 (PMC11929985; doi:10.1186/s12889-025-21728-1)
Supplement: Supplementary file 1 — Additional file 1. Derivation of variables. [file 12889_2025_21728_MOESM1_ESM.docx]

## Supplementary data

## Additional file 1 Derivation of variables

Records from the CPRD clinical, referral, therapy and test files and linked practice-level Indices of multiple deprivation (IMD) data were used to derive sociodemographic and health-related variables used in the analysis, using pre-specified code lists. Where possible, code lists were adapted from previously published lists, and updated where required using the CPRD GOLD code browser (2020) and clinical input.

These include

**sociodemographic variables**: age, ethnic group, and practice-level IMD quintiles (as a proxy for women’s socioeconomic circumstances);

**health risk factor**: BMI and smoking;

**medical conditions prior to pregnancy**: diabetes mellitus, hypertension, asthma, epilepsy, cardiovascular disease (CVD), common mental disorders (depression and/or anxiety), and polycystic ovary syndrome (PCOS). These conditions were selected because they are recognised risk factors for poor pregnancy, perinatal and longer-term outcomes, particularly if severe or poorly managed, and also because their inclusion in the Quality Outcomes Framework means that they should be more reliably monitored and recorded.

**pre-pregnancy care (PPC) and advice**: details see Appendix 2.

**women’s interaction with the GP services**: face-to-face contact with GP and/or nurse and smear test/cytology appointments (detailed code list see Appendix 2) were derived to explore opportunities for GPs to intervene before pregnancy starts.

**active management of medical conditions:** additionally, we explored ways to capture appropriate management of these existing health conditions, e.g. HbA1c levels for DM, blood pressure monitoring for hypertension, appropriate check-ups or reviews for asthma and epilepsy, and derived relevant indicators for active management of these conditions. Detailed code list see Appendix 3.

| **Variable** | **Categories** | **Derivation** | **Sources of code list and/or references** |
| --- | --- | --- | --- |
| **Sociodemographic characteristics** |  |  |  |
| **Maternal age** | <20 yrs  20-24 yrs  25-29 yrs  30-34 yrs  35-39 yrs  40-44 yrs  ≥45 yrs | • For age on 1^st^ Jan 2017, difference between 1^st^ Jan 2017 and mother’s estimated date of birth in the CPRD GOLD (set to 15^th^ day of the month when the date is missing and set to 30^th^ June of their year of birth when both date and month are missing, as CPRD does not provide exact date of birth for women) was calculated.  • For maternal age at the beginning of the pregnancy, years between the beginning of the pregnancy and mother’s estimated date of birth was calculated. | N/A |
| **Ethnic group** | White British  White other  Mixed  Asian or Asian British  Black or Black British  Chinese or other  *Missing* | We used the ‘medcode’ field of the CPRD GOLD Clinical data file to identify ethnicity codes.^1^ A 17-level categorisation in the 2011 Census for England and Wales was created based on the most recent ethnic code before 1st Jan 2017 for all women of reproductive age, before being collapsed into a 6-level categorisation variable used in the analysis. Similar approach was taken to create the ethnic group variable using the most recent ethnic code before the start of the pregnancy for those whose pregnancy started in 2017-2018. | Mathur R, Bhaskaran K, Chaturvedi N, Leon DA, vanStaa T, Grundy E, et al. Completeness and usability of ethnicity data in UK-based primary care and hospital databases. *J Public Health (Oxf)*. 2014;**36**(4):684-92. |
| **Practice IMD** | 1 (least deprived)  2  3  4  5 | Existing variable in the linked IMD dataset provided by CPRD. | N/A |
| **Health status and risk behaviours** |  |  |  |
| **BMI (kg/m^2^)** | <18.5  18.5-24.9  25-29.9  ≥30  *Missing* | Derived based on an algorithm as described in the reference.^2^ Briefly, We used either the most recently recorded BMI or derived from the most recent height and weight measurement records in CPRD to calculated BMI (BMI=weight/height^2^). Records without any measurements or with implausible measurements were excluded. | Bhaskaran K, Forbes HJ, Douglas I, Leon DA, Smeeth L. Representativeness and optimal use of body mass index (BMI) in the UK Clinical Practice Research Datalink (CPRD). *BMJ Open*. 2013;**3**(9):e003389. |
| **Smoking** | Never smokers  Former smokers  Current smokers  *Missing* | Derived based on entity type for smoking, and code lists created by search and based on paper by Stocks et al.^3^ | Stocks SJ, Kontopantelis E, Akbarov A, Rodgers S, Avery AJ, Ashcroft DM. Examining variations in prescribing safety in UK general practice: cross sectional study using the Clinical Practice Research Datalink. *BMJ*. 2015;**351**:h5501.  [**https://clinicalcodes.rss.mhs.man.ac.uk/medcodes/article/25/codelist/res25-p18_smoking_status/**](https://clinicalcodes.rss.mhs.man.ac.uk/medcodes/article/25/codelist/res25-p18_smoking_status/) |
| **Pre-existing chronic health conditions** |  |  |  |
| **Diabetes mellitus** | Yes/No | We used the ‘medcode’ field of the CPRD GOLD Clinical and Referral data files to identify codes related to diabetes mellitus based on code lists in the Cambridge CPRD code depository and the LSHTM Data Compass depository. | Cambridge CPRD code depository  <https://www.phpc.cam.ac.uk/pcu/research/research-groups/crmh/cprd_cam/>  LSHTM Data Compass CPRD code depository <https://datacompass.lshtm.ac.uk/id/eprint/3495/> |
| **Hypertension** | Yes/No | We used the ‘medcode’ field of the CPRD GOLD Clinical and Referral data files to identify codes related to hypertension based on code lists in the Cambridge CPRD code depository. | Cambridge CPRD code depository  <https://www.phpc.cam.ac.uk/pcu/research/research-groups/crmh/cprd_cam/> |
| **Asthma** | Yes/No | We used the ‘medcode’ field of the CPRD GOLD Clinical and Referral data files, and ‘prodcode’ field of the CPRD GOLD Therapy data file to identify codes related to asthma based on code lists in the Cambridge CPRD code depository and paper by Nissen et al.^4^ | Cambridge CPRD code depository <https://www.phpc.cam.ac.uk/pcu/research/research-groups/crmh/cprd_cam/>  Nissen F, Morales DR, Mullerova H, Smeeth L, Douglas IJ, Quint JK. Validation of asthma recording in the Clinical Practice Research Datalink (CPRD). *BMJ Open*. 2017;**7**(8):e017474. |
| **Actively managed asthma (ever diagnosed+treated in the last year)** | Yes/No | Derived using the same codes and with similar approaches as described for pre-existing asthma above. Actively managed asthma is defined as having ever been diagnosed and treated in the last year. | Same as asthma. |
| **Epilepsy** | Yes/No | We used the ‘medcode’ field of the CPRD GOLD Clinical and Referral data files, and ‘prodcode’ field of the CPRD GOLD Therapy data file to identify codes related to epilepsy based on code lists in the Cambridge CPRD code depository, the LSHTM Data Compass depository and paper by Lee et al^5^ with input from clinicians. | Cambridge CPRD code depository <https://www.phpc.cam.ac.uk/pcu/research/research-groups/crmh/cprd_cam/>  LSHTM Data Compass CPRD code depository  <https://datacompass.lshtm.ac.uk/id/eprint/864/>  Lee SI, Azcoaga-Lorenzo A, Agrawal U, Kennedy JI, Fagbamigbe AF, Hope H, et al. Epidemiology of pre-existing multimorbidity in pregnant women in the UK in 2018: a population-based cross-sectional study. *BMC Pregnancy Childbirth*. 2022;**22**(1):120. |
| **Actively managed epilepsy (ever diagnosed+treated in the last year)** | Yes/No | Derived using the same codes and with similar approaches as described for pre-existing epilepsy above. Actively managed epilepsy is defined as having ever been diagnosed and treated in the last year. | Same as epilepsy. |
| **Cardiovascular disease (CVD)** | Yes/No | We used the ‘medcode’ field of the CPRD GOLD Clinical and Referral data files to identify codes related to CVD based on code lists in the Cambridge CPRD code depository. | Cambridge CPRD code depository <https://www.phpc.cam.ac.uk/pcu/research/research-groups/crmh/cprd_cam/> |
| **Common mental disorders (depression and/or anxiety)** | Yes/No | Derived based on an algorithm and code lists as described in the reference.^6^ | Tianyi FL, Li Y, Alderdice F, Quigley MA, Kurinczuk JJ, Bankhead C, et al. The association between conception history and subsequent postpartum depression and/or anxiety: Evidence from the Clinical Practice Research Datalink 1991-2013. *J Affect Disord*. 2022;**310**:266-73. |
| **Polycystic ovary syndrome (PCOS)** | Yes/No | We used the ‘medcode’ field of the CPRD GOLD Clinical and Referral data files to identify codes related to PCOS based on code lists in the paper by Subramanian et al.^7^ | Subramanian A, Lee SI, Phillips K, Toulis KA, Kempegowda P, O'Reilly MW, et al. Polycystic ovary syndrome and risk of adverse obstetric outcomes: a retrospective population-based matched cohort study in England. *BMC Med*. 2022;**20**(1):298. |

**References**

1. Mathur R, Bhaskaran K, Chaturvedi N, Leon DA, vanStaa T, Grundy E, et al. Completeness and usability of ethnicity data in UK-based primary care and hospital databases. *J Public Health (Oxf)*. 2014;**36**(4):684-92.

2. Bhaskaran K, Forbes HJ, Douglas I, Leon DA, Smeeth L. Representativeness and optimal use of body mass index (BMI) in the UK Clinical Practice Research Datalink (CPRD). *BMJ Open*. 2013;**3**(9):e003389.

3. Stocks SJ, Kontopantelis E, Akbarov A, Rodgers S, Avery AJ, Ashcroft DM. Examining variations in prescribing safety in UK general practice: cross sectional study using the Clinical Practice Research Datalink. *BMJ*. 2015;**351**:h5501.

4. Nissen F, Morales DR, Mullerova H, Smeeth L, Douglas IJ, Quint JK. Validation of asthma recording in the Clinical Practice Research Datalink (CPRD). *BMJ Open*. 2017;**7**(8):e017474.

5. Lee SI, Azcoaga-Lorenzo A, Agrawal U, Kennedy JI, Fagbamigbe AF, Hope H, et al. Epidemiology of pre-existing multimorbidity in pregnant women in the UK in 2018: a population-based cross-sectional study. *BMC Pregnancy Childbirth*. 2022;**22**(1):120.

6. Tianyi FL, Li Y, Alderdice F, Quigley MA, Kurinczuk JJ, Bankhead C, et al. The association between conception history and subsequent postpartum depression and/or anxiety: Evidence from the Clinical Practice Research Datalink 1991-2013. *J Affect Disord*. 2022;**310**:266-73.

7. Subramanian A, Lee SI, Phillips K, Toulis KA, Kempegowda P, O'Reilly MW, et al. Polycystic ovary syndrome and risk of adverse obstetric outcomes: a retrospective population-based matched cohort study in England. *BMC Med*. 2022;**20**(1):298.
