# Supplementary material for: Pre-pregnancy care in general practice in England: cross-sectional observational study using administrative routine health data
Source: BMC Public Health. 2025 Mar 22;25:1101. doi: 10.1186/s12889-025-21728-1 (PMC11929985; doi:10.1186/s12889-025-21728-1)
Supplement: Supplementary file 2 — Additional file 2. Identification of pre-pregnancy care and advice. [file 12889_2025_21728_MOESM2_ESM.docx]

## Additional file 2 Identification of pre-pregnancy care and advice

To capture pre-pregnancy care (PPC) and advice, records were restricted to the year prior to the start of the pregnancy for those who became pregnant between 2017 and 2018. For women without a pregnancy record, the year before the baseline date (01/01/2017) was used. Diagnostic and symptom codes relating to PPC were extracted from the women’s Clinical and Referral records in CPRD GOLD using pre-specified code lists using the CPRD GOLD code browser (2020) and clinical input. Records of referrals to secondary and tertiary care were also searched to identify care provided by other services.

Two types of care were considered pre-pregnancy related:

1. **specific PPC** including three subtypes: **folic acid advice, fertility/infertility discussions and general PPC and advice**.
2. **general health promotion** including five subtypes: **nutrition, smoking cessation, weight management, alcohol drinking advice, family planning and contraception**.

Additionally, we explored **opportunities** for PPC related discussion**, i.e. smear test/cytology appointments.**

| **Categories of codes related to pre-pregnancy care and advice** |
| --- |
| 1 Specific preconception codes |
| 2 Specific preconception codes to be combined with time |
| 3 Individual aspects of general health promotion |
| **4 Opportunities (smear test/cytology appointments)** |

**Specific PPC**

**Code list for folic acid advice**

| **medcode** | **readcode** | **Read term** |
| --- | --- | --- |
| 21532 | 6760 | Folic acid advice - pre-pregnancy |
| 40842 | 8BP2.00 | Over the counter folic acid therapy |
| 96922 | 8B6Y.00 | Folic acid prophylaxis |
| 30269 | ZC61Y00 | Folic acid supplementation |
| 97034 | 67AE.00 | Folic acid advice in first trimester of pregnancy |
| 18580 | ZC61Y11 | Folate supplementation |

**Code list for fertility/infertility discussions**

| **medcode** | **readcode** | **Read term** |
| --- | --- | --- |
| 52132 | K5B0000 | Primary anovulatory infertility |
| 36264 | K26y200 | Infertility due to efferent duct obstruction |
| 60861 | K5B2000 | Primary tubal infertility |
| 48461 | K5B5.00 | Female infertility of vaginal origin |
| 104569 | K5B7.00 | Female infertility due to diminished ovarian reserve |
| 69884 | K5B1.00 | Female infertility of pituitary - hypothalamic origin |
| 2949 | 9N1y600 | Seen in fertility clinic |
| 4571 | 9N07.00 | Seen in fertility clinic |
| 61299 | K5B3.00 | Female infertility of uterine origin |
| 99535 | K5B1100 | Secondary pituitary - hypothalamic infertility |
| 1943 | K5By100 | Secondary infertility unspecified |
| 69751 | ZV26z00 | [V]Unspecified infertility management |
| 26088 | ZV26200 | [V]Infertility investigation and testing |
| 9036 | 6778.11 | Fertility counselling |
| 50116 | K5B0100 | Secondary anovulatory infertility |
| 99069 | Kyu9G00 | [X]Female infertility of other origin |
| 45985 | K5B2100 | Secondary tubal infertility |
| 9983 | 8C8Z.00 | Treatment for infertility NOS |
| 4977 | K5B0.00 | Female infertility of anovulatory origin |
| 35074 | K5B2.00 | Female infertility of tubal origin |
| 5239 | 8HTB.00 | Referral to fertility clinic |
| 73151 | K5B3100 | Secondary uterine infertility |
| 94448 | K5B1000 | Primary pituitary - hypothalamic infertility |
| 113724 | K26y000 | Infertility due to drug therapy |
| 33458 | 8C82.00 | Female infertility therapy |
| 1810 | 8C8..00 | Treatment for infertility |
| 9133 | 7E29000 | Fertility investigation of female NEC |
| 25077 | K5B2z00 | Female infertility of tubal origin NOS |
| 2548 | 6778 | Procreat/fertility counselling |
| 39295 | ZV26400 | [V]Infertility general advice and counselling |
| 1154 | 3189 | Infertility investigations NOS |
| 36458 | K5By.00 | Other female infertility |
| 102589 | 8Cf..00 | Infertility care |
| 63421 | K5B0z00 | Female infertility of anovulatory origin NOS |
| 16131 | 3189.11 | Infertility investigation -fem |
| 30392 | K5Bz.00 | Female infertility NOS |
| 101598 | K26y300 | Infertility due to radiation |
| 41692 | 3189100 | Female infertility test abnormal |
| 53018 | K5Byz00 | Other female infertility NOS |
| 62698 | K5B4.00 | Female infertility of cervical origin |
| 54282 | K5B6.00 | Female infertility associated with male factors |
| 91280 | K5B3000 | Primary uterine infertility |
| 17756 | 3189000 | Female infertility test normal |
| 32199 | 1597.11 | H/O: female infertility |
| 69324 | K5B4100 | Secondary cervical infertility |
| 2957 | 1AZ2.11 | Infertility problem |
| 108577 | K26y400 | Infertility in systemic disease |
| 2014 | K5By000 | Primary infertility unspecified |
| 1808 | K5B..00 | Infertility - female |
| 45005 | 1AZ5.00 | Fertility problems in partner |
| 25361 | Z4P1.00 | Fertility counselling |
| 7246 | K5Byz11 | Subfertility |
| 62084 | K5B1z00 | Female infertility of pituitary - hypothalamic cause NOS |
| 40072 | 4Z0..00 | Infertility studies |
| 96463 | K5B5100 | Secondary vaginal infertility |
| 10445 | ZG91.00 | Advice on fertility and infertility |
| 68664 | K5B3z00 | Female infertility of uterine origin NOS |
| 16376 | 1597 | H/O: infertility - female |
| 97461 | K5B4000 | Primary cervical infertility |
| 7351 | 1AZ2.00 | Fertility problem |
| 111013 | K5B5z00 | Female infertility of vaginal origin NOS |
| 111102 | K5B5000 | Primary vaginal infertility |
| 26150 | ZV26y00 | [V]Other specified infertility management |
| 9938 | ZV26.00 | [V]Infertility management |
| 37047 | 6242 | A/N care: H/O infertility |
| 52685 | ZV23000 | [V]Pregnancy with history of infertility |

**Code list for general pre-pregnancy care and advice**

| **medcode** | **readcode** | **Read term** |
| --- | --- | --- |
| 10205 | ZG9..00 | Advice relating to pregnancy and fertility |
| 49884 | 6761 | Diabetic pre-pregnancy counselling |
| 4609 | 676..00 | Pre-pregnancy counselling |
| 102767 | 67IJ100 | Pre-conception advice for diabetes mellitus |
| 102264 | 8IB3.00 | Pre-conception advic fr patients with epilepsy not indicated |
| 10761 | 67IJ.00 | Pre-conception advice |
| 41319 | Z211.00 | Preconception care |
| 100920 | 67IJ000 | Pre-conception advice for patients with epilepsy |
| 50937 | 8HTe.00 | Referral to diabetes preconception counselling clinic |
| 12996 | 6125 | Trying to conceive |
| 12997 | 6125.11 | Planning to start family |
| 36903 | 67AZ.00 | Pregnancy advice NOS |
| 36235 | 67A4.00 | Pregnancy exercise advice |
| 102359 | 67AF.00 | Pregnancy advice for patients with epilepsy |
| 102375 | 8IB4.00 | Pregnancy advice for patients with epilepsy not indicated |
| 68325 | 8CAW.00 | Patient advised to have pregnancy test |
| 35859 | 67A5.00 | Pregnancy alcohol advice |
| 38556 | 67A7.00 | Pregnancy dental advice |
| 5778 | 67A..00 | Pregnancy advice |
| 10184 | 67A3.00 | Pregnancy smoking advice |
| 43140 | 67A2.00 | Diet in pregnancy advice |
| 30351 | 67A6.00 | Drugs in pregnancy advice |
| 104705 | 8CL4.00 | Discussion about ectopic pregnancy risk |
| 104542 | 67AH.00 | Education about toxoplasmosis precautions during pregnancy |
| 12874 | Z22C600 | Number of previous pregnancies |
| 16706 | ZV72400 | [V]Pregnancy examination or test, pregnancy unconfirmed |
| 50908 | L242.00 | Uterine scar from previous surgery in pregnancy/childb/puerp |
| 32368 | Z22C400 | Duration of pregnancy at time of previous miscarriage |
| 113236 | L228100 | Multiple pregnancy with malpresentation - delivered |
| 9986 | Z212.11 | Pregnancy care |
| 25254 | Z21..00 | Care relating to reproduction and pregnancy |
| 24944 | L163300 | Pregnancy care of habitual aborter |
| 55618 | ZV23600 | [V]Supervisn/pregnancy wth history insufficnt antenatal care |
| 30365 | Z22AA00 | Wanted pregnancy |
| 15065 | 8B7..11 | Pregnancy vitamin/iron prophyl |
| 29746 | 8B75.00 | Vitamin supplement - pregnancy |
| 10775 | 8B74.00 | Iron supplement in pregnancy |
| 108013 | ZC2CB00 | Dietary advice for gestational diabetes |
| 43140 | 67A2.00 | Diet in pregnancy advice |
| 10184 | 67A3.00 | Pregnancy smoking advice |
| 35859 | 67A5.00 | Pregnancy alcohol advice |

**General health promotion**

**Code list for nutrition**

| **medcode** | **readcode** | **Read term** |
| --- | --- | --- |
| 106408 | 67DE.00 | Provision of information about vitamin D supplementation |
| 108038 | 8CA4N11 | Vitamin advice |
| 28723 | ZC61b00 | Calcium - dietary supplementation |
| 51996 | ZC2B.00 | Advice to change vitamin intake |
| 33010 | ZC61W00 | Water soluble vitamin supplementation |
| 34033 | ZC61Z00 | Fat soluble vitamin supplementation |
| 107589 | 8CA4N00 | Vitamin education |
| 5711 | 8B7..12 | Vitamin prophylaxis |
| 40872 | ZC61X00 | Vitamin supplementation |
| 9918 | ZC63.00 | Nutritional supplementation |
| 108349 | 8CA4X00 | Recommendation to take oral nutritional supplements |
| 109594 | 8CA4X11 | Advice to take oral nutritional supplements |
| 19166 | ZC61.00 | Nutrient supplementation |
| 10819 | ZC61a00 | Mineral supplementation |
| 28439 | ZC62.00 | Nutritional product supplementation |
| 101801 | 66At100 | Type II diabetic dietary review |
| 26007 | ZC35.00 | Dietary intake assessment using food diary |
| 35405 | ZRBB.11 | DINE-Diet instr nutr educ ques |
| 13076 | 13A3.00 | Weight reducing diet |
| 69176 | ZC2..11 | Advice on diet |
| 69175 | ZC2..12 | Advice about diet |
| 55085 | ZC36.00 | Diet in ass usng food fr quest |
| 46950 | ZC34.00 | Weighed dietary intake assessment |
| 108548 | 8CA4V11 | Dietary advice to maximise nutritional intake |
| 102345 | 1FH..00 | Healthy diet |
| 102704 | 66At000 | Type I diabetic dietary review |
| 25041 | ZC2CA00 | Dietary advice for type II diabetes |
| 48971 | ZC32.00 | Dietary history assessment using written intake record |
| 31922 | ZC4..00 | Dietary health promotion advice |
| 2803 | 8CA4.00 | Patient advised re diet |
| 109205 | 8CA4V00 | Dietary education to maximise nutritional intake |
| 45807 | ZV65314 | [V]Dietary surveillance |
| 16881 | ZV65312 | [V]Dietary counselling in diabetes mellitus |
| 35918 | ZV65313 | [V]Dietary counselling |
| 13080 | 1FA..00 | Diet good |
| 8304 | ZC2C711 | Dietary advice for weight reduction |
| 11636 | 8CA4z00 | Pt advised re diet NOS |
| 13078 | 13AC.00 | Diabetic weight reducing diet |
| 13069 | 66A8.00 | Has seen dietician - diabetes |
| 68776 | ZRBB.00 | Dietary instrument for nutrition education questionnaire |
| 17477 | ZV65319 | [V]Dietary counselling in obesity |
| 101177 | 66At.00 | Diabetic dietary review |
| 11458 | 8CA4000 | Pt advised re wt reducing diet |
| 98146 | 67H7.00 | Lifestyle advice regarding diet |
| 104453 | 66At011 | Type 1 diabetic dietary review |
| 43657 | 8B9Z.00 | Dietary prophylaxis NOS |
| 17616 | ZV65300 | [V]Dietary surveillance and counselling |
| 26604 | 66AY.00 | Diabetic diet - good compliance |
| 17643 | ZV4K300 | [V]Inappropriate diet and eating habits |
| 8964 | 8B57.00 | Weight reducing diet |
| 8414 | 8CA4100 | Pt advised re diabetic diet |
| 13074 | 13B1.00 | Diabetic diet |
| 38078 | 66A9.00 | Understands diet - diabetes |
| 101217 | 66CR.00 | Int risk health ass overwt ob advice about diet physical act |
| 12656 | 1FB..00 | Diet poor |
| 10642 | ZC2C800 | Dietary advice for diabetes mellitus |
| 109878 | ZC2C911 | Diet advice for insulin-dependent diabetes |
| 1661 | 6799 | Health ed. - diet |
| 11763 | ZC2C700 | Patient advised about weight-reducing diet |
| 3176 | 66C4.00 | Has seen dietician - obesity |
| 10728 | ZC2CM00 | Dietary advice for obesity |
| 21689 | 13AB.00 | Diabetic lipid lowering diet |
| 25636 | 66Aa.00 | Diabetic diet - poor compliance |
| 7563 | 66A3.00 | Diabetic on diet only |
| 104887 | 66CS.00 | Inter risk hlth overwght obesity adv diet phys act cons drug |
| 28733 | ZQ3L.00 | Dietary intake assessment |
| 17507 | ZC31.00 | Review of current diet |
| 102611 | 66At111 | Type 2 diabetic dietary review |
| 17682 | 8CE4.00 | Diet leaflet given |
| 69043 | ZC2C900 | Dietary advice for type I diabetes |
| 61420 | ZC2CN00 | Dietary advice for weight gain |
| 33537 | 8B9..00 | Dietary prophylaxis |
| 6397 | 8B5..00 | Dietary regime |
| 42898 | ZC31.11 | Dietary history assessment using recall |
| 31067 | ZC2..00 | Dietary advice |
| 43375 | ZC2CO00 | Dietary advice for weight loss |
| 48757 | ZC33.00 | Dietary intake assessment using food models |
| 31989 | ZC3..00 | Dietary intake assessment procedures |
| 110393 | 13B1000 | Diabetic carbohydrate counting diet |
| 95561 | ZA11925 | Advice on diet |

**Code list for smoking cessation**

| **medcode** | **readcode** | **Read term** |
| --- | --- | --- |
| 10615 | 9OO8.00 | Stop smoking monitor phone inv |
| 14829 | 137C.00 | Keeps trying to stop smoking |
| 465369 | 137K.00 | Stopped smoking |
| 41159 | 8HkQ.00 | Referral to NHS stop smoking service |
| 4921 | 9OO6.00 | Stop smoking monitor 3rd lettr |
| 100687 | 9OO4.00 | Stop smoking monitor 1st lettr |
| 19957 | 137b.00 | Ready to stop smoking |
| 14367 | 9OO2.00 | Refuses stop smoking monitor |
| 108 | 9OO9.00 | Stop smoking monitoring delete |
| 93297 | 8HTK.00 | Referral to stop-smoking clinic |
| 46600 | 9OO..12 | Stop smoking monitoring admin. |
| 10547 | 9OO..11 | Stop smoking clinic admin. |
| 8190 | 8CdB.00 | Stop smoking service opportunity signposted |
| 2941 | 9OO7.00 | Stop smoking monitor verb.inv. |
| 14759 | 9OO5.00 | Stop smoking monitor 2nd lettr |
| 4220 | 137K000 | Recently stopped smoking |
| 292 | 9OOB100 | Stop smoking invitation second SMS text message |
| 2387 | 9OO3.00 | Stop smoking monitor default |
| 80149 | 9OO1.00 | Attends stop smoking monitor. |
| 4282 | 9OOB.00 | Stop smoking invitation short message service text message |
| 3873 | 9OOB000 | Stop smoking invitation first SMS text message |
| 23973 | 137c.00 | Thinking about stopping smoking |
| 16149 | 9OOZ.00 | Stop smoking monitor admin.NOS |
| 16275 | 9OOA.00 | Stop smoking monitor.chck done |
| 1866 | 8HBM.00 | Stop smoking face to face follow-up |
| 136 | 9OOB200 | Stop smoking invitation third SMS text message |
| 209306 | 8H7i.00 | Referral to smoking cessation advisor |
| 28 | 9NdV.00 | Consent given follow-up after smoking cessation intervention |
| 5125 | 13p5000 | Practice based smoking cessation programme start date |
| 9215 | 745Hz00 | Smoking cessation therapy NOS |
| 115088 | 745H.00 | Smoking cessation therapy |
| 13839 | 745H400 | Smoking cessation drug therapy |
| 14395 | 8T08.00 | Referral to smoking cessation service |
| 13752 | 9NS0200 | Referral for smoking cessation service offered |
| 2267 | 9kc..00 | Smoking cessation - enhanced services administration |
| 1925 | 9Ndf.00 | Consent given for follow-up by smoking cessation team |
| 3266 | 745Hy00 | Other specified smoking cessation therapy |
| 24468 | 67H6.00 | Brief intervention for smoking cessation |
| 636 | 9NdW.00 | Consent given for smoking cessation data sharing |
| 2 | 9kc0.11 | Smoking cessation ESA monitoring template completed |
| 154831 | 13p..00 | Smoking cessation milestones |
| 35165 | 13p5.00 | Smoking cessation programme start date |
| 10868689 | 8CAL.00 | Smoking cessation advice |
| 305709 | 9N2k.00 | Seen by smoking cessation advisor |
| 85586 | 13p0.00 | Negotiated date for cessation of smoking |
| 1535 | 8CAg.00 | Smoking cessation advice provided by community pharmacist |
| 1624 | 9km..11 | Ex-smoker annual review |
| 31323 | 9OO..00 | Anti-smoking monitoring admin. |
| 192 | 9kc0.00 | Smoking cessatn monitor template complet - enhanc serv admin |
| 11036 | ZG23300 | Advice on smoking |
| 241390 | 137G.00 | Trying to give up smoking |
| 63778 | 67H1.00 | Lifestyle advice regarding smoking |
| 7442 | 137V.00 | Smoking reduced |
| 75147 | 137T.00 | Date ceased smoking |
| 47 | 9kn..11 | Non-smoker annual review |
| 12657 | 13p4.00 | Smoking free weeks |
| 6858 | 13p2.00 | Smoking status between 4 and 52 weeks |
| 38122 | 13p1.00 | Smoking status at 4 weeks |
| 3613105 | 6791 | Health ed. - smoking |
| 4978 | 9ko..11 | Current smoker annual review |
| 36231 | 68T..00 | Tobacco usage screen |
| 16096 | 6893 | Tobacco usage screen |

**Code list for** **weight management**

| **medcode** | **readcode** | **Read term** |
| --- | --- | --- |
| 23376 | 22A3.00 | O/E - weight within 10% ideal |
| 102852 | 9NiX.00 | DNA weight management special interest GP clinic |
| 13076 | 13A3.00 | Weight reducing diet |
| 21520 | 22AZ.00 | O/E - weight NOS |
| 32974 | 22A5.00 | O/E - weight > 20% over ideal |
| 42309 | 22A7.00 | Baseline weight |
| 26355 | 66C9.00 | Target weight discussed |
| 104002 | 22A9.00 | Percentage weight loss |
| 40640 | 66CF.00 | Target weight |
| 22142 | ZG53.00 | Advice about weight |
| 6713 | 8CA4011 | Patient advised to lose weight |
| 34363 | 1621.11 | Weight static |
| 102631 | 8CP5.00 | Discussion about weight management programme |
| 102566 | 9NS0300 | Referral to weight management service offered |
| 35407 | 66CJ.00 | Weight management plan completed |
| 8304 | ZC2C711 | Dietary advice for weight reduction |
| 13078 | 13AC.00 | Diabetic weight reducing diet |
| 3647 | R032.00 | [D]Abnormal loss of weight |
| 103499 | 22AA.00 | Overweight |
| 112889 | 66CF400 | Target weight to achieve ten percent weight loss |
| 107431 | 9N1yK00 | Seen in weight management clinic |
| 26182 | 66CH.00 | Weight management plan started |
| 102514 | 66CN.00 | Risk health associated overweight and obesity, at high risk |
| 26415 | 66CA.00 | Ideal weight discussed |
| 19208 | 1624 | Abnormal weight gain |
| 102645 | 8HHH000 | Referral to local authority weight management programme |
| 11882 | 67I9.00 | Advice about weight |
| 12445 | ZG53100 | Patient advised to lose weight |
| 2 | 22A..00 | O/E - weight |
| 5812 | 1625.11 | Abnormal weight loss - symptom |
| 12398 | 1D1A.00 | Complaining of weight loss |
| 105396 | 8H4n.00 | Referral to weight management special interest GP |
| 108608 | 66Cb.00 | Intensive weight management programme commenced |
| 12530 | R034800 | [D]Underweight |
| 32879 | 66CG.00 | Weight management programme offered |
| 96348 | 6B4..11 | Counterweight programme |
| 8964 | 8B57.00 | Weight reducing diet |
| 654 | 1623 | Weight decreasing |
| 111737 | 66CF100 | 6 month target weight |
| 6533 | R031.00 | [D]Abnormal weight gain |
| 110708 | 8HHH100 | Referral to residential weight management programme |
| 13056 | 679P.00 | Health education - weight management |
| 106010 | 66CP.00 | Risk health associ overweight and obesity, at very high risk |
| 102150 | 66CM.00 | Risk health associ overweight and obesity, at increased risk |
| 109470 | 66CF000 | 3 month target weight |
| 9015 | 22K4.00 | Body mass index index 25-29 - overweight |
| 4663 | 1625 | Abnormal weight loss |
| 102563 | 1627 | Unintentional weight loss |
| 8041 | 66C..11 | Weight monitoring |
| 110331 | 66Cc.00 | Intensive weight management programme ended |
| 17949 | 6878.11 | Weight screen |
| 103849 | 8CdC.00 | Weight management service opportunity signposted |
| 11763 | ZC2C700 | Patient advised about weight-reducing diet |
| 103926 | 9c01000 | Expectation of weight management programme |
| 29029 | 22A2.00 | O/E -weight 10-20% below ideal |
| 16404 | 22A4.00 | O/E - weight 10-20% over ideal |
| 108351 | 1629 | Unintentional weight gain |
| 25061 | 66CB.00 | Ideal weight |
| 61420 | ZC2CN00 | Dietary advice for weight gain |
| 113461 | 66CF200 | 12 month target weight |
| 126 | 22A6.00 | O/E - Underweight |
| 26473 | 22A1.00 | O/E - weight > 20% below ideal |
| 3355 | 66C9.11 | Weight loss advised |
| 25951 | 8HHH.00 | Refer to weight management programme |
| 94885 | 6B4..00 | Counterweight weight management programme |
| 8481 | 66CC.00 | Wants to lose weight |
| 43375 | ZC2CO00 | Dietary advice for weight loss |
| 105170 | 67K9.00 | Cycle of change stage, weight management |
| 94788 | 8B5B.00 | Weight gain diet |
| 101043 | 8Cd7.00 | Advice given about weight management |
| 2839 | 22A4.11 | O/E - overweight |
| 104724 | 66CL.00 | Risk health associa overweight obesity, at no increased risk |
| 22343 | ZC17.00 | Exercising to lose weight |
| 108478 | 22KE.00 | Obese class III (BMI equal to or greater than 40.0) |
| 70898 | C38z.00 | Obesity and other hyperalimentation NOS |
| 17897 | ZV77800 | [V]Screening for obesity |
| 21744 | 9OK..11 | Obesity clinic administration |
| 40153 | 66CZ.00 | Obesity monitoring NOS |
| 55585 | 9OK6.00 | Obesity monitoring 3rd letter |
| 52036 | 9OK3.00 | Obesity monitoring default |
| 73304 | 9OK9.00 | Obesity monitoring deleted |
| 27570 | 66C7.00 | Treatment of obesity stopped |
| 104129 | C380600 | Adult-onset obesity |
| 49250 | C380100 | Drug-induced obesity |
| 108147 | 8T11.00 | Referral to multidisciplinary obesity clinic |
| 38799 | C380000 | Obesity due to excess calories |
| 59780 | 222A.00 | O/E - obese |
| 17477 | ZV65319 | [V]Dietary counselling in obesity |
| 110415 | 66CX.00 | Obesity multidisciplinary case review |
| 52034 | 9OK1.00 | Attends obesity monitoring |
| 70950 | 9OK7.00 | Obesity monitoring verbal inv. |
| 49409 | 9OK4.00 | Obesity monitoring 1st letter |
| 104421 | C380700 | Lifelong obesity |
| 17470 | 6878 | Obesity screen |
| 17444 | 66CE.00 | Reason for obesity therapy - occupational |
| 66406 | C38..00 | Obesity and other hyperalimentation |
| 22695 | C380400 | Central obesity |
| 11461 | 66C..00 | Obesity monitoring |
| 69757 | Cyu7000 | [X]Other obesity |
| 22556 | 22K7.00 | Body mass index 40+ - severely obese |
| 108610 | 22KD.00 | Obese class II (body mass index 35.0 - 39.9) |
| 29538 | 66C2.00 | Follow-up obesity assessment |
| 38632 | 66C6.00 | Treatment of obesity started |
| 25968 | C380500 | Generalised obesity |
| 52782 | Cyu7.00 | [X]Obesity and other hyperalimentation |
| 103574 | C38y011 | Obesity hypoventilation syndrome |
| 3176 | 66C4.00 | Has seen dietician - obesity |
| 10728 | ZC2CM00 | Dietary advice for obesity |
| 104887 | 66CS.00 | Inter risk hlth overwght obesity adv diet phys act cons drug |
| 110196 | 66Ce.00 | Telehealth obesity monitoring |
| 13278 | 22K5.00 | Body mass index 30+ - obesity |
| 38059 | C380200 | Extreme obesity with alveolar hypoventilation |
| 7984 | 22A5.11 | O/E - obese |
| 52735 | 9OKZ.00 | Obesity monitoring admin.NOS |
| 47439 | 9OKA.00 | Obesity monitoring check done |
| 11401 | C38z000 | Simple obesity NOS |
| 64712 | 66C5.00 | Treatment of obesity changed |
| 38658 | 66C1.00 | Initial obesity assessment |
| 55586 | 9OK5.00 | Obesity monitoring 2nd letter |
| 430 | C380.00 | Obesity |
| 108355 | 8CV7.00 | Anti-obesity drug therapy commenced |
| 32843 | 9OK..00 | Obesity monitoring admin. |
| 67517 | 9OK8.00 | Obesity monitor phone invite |
| 108694 | 22KC.00 | Obese class I (body mass index 30.0 - 34.9) |
| 8854 | C380300 | Morbid obesity |
| 107231 | 22KA.00 | Target body mass index |
| 8105 | 22K..00 | Body Mass Index |
| 44291 | 22K8.00 | Body mass index 20-24 - normal |
| 24496 | 22K6.00 | Body mass index less than 20 |
| 32914 | 22K3.00 | Body Mass Index low K/M2 |
| 101047 | 22K9.00 | Body mass index centile |
| 28937 | 22K2.00 | Body Mass Index high K/M2 |
| 28946 | 22K1.00 | Body Mass Index normal K/M2 |
| 105791 | 22K9000 | Baseline body mass index centile |
| 105800 | 22KB.00 | Baseline body mass index |

**Code list for alcohol drinking advice**

| **medcode** | **readcode** | **Read term** |
| --- | --- | --- |
| 11491 | 6792 | Health ed. - alcohol |
| 18711 | 67H0.00 | Lifestyle advice regarding alcohol |
| 12982 | 136K.00 | Alcohol intake above recommended sensible limits |
| 35330 | 9k11.00 | Alcohol consumption counselling |
| 48545 | 9k13.00 | Alcohol questionnaire completed |
| 54209 | ZC2H.00 | Advice to change alcohol intake |
| 61383 | Z191200 | Planned reduction of alcohol consumption |
| 7692 | 8CAM.00 | Patient advised about alcohol |
| 26472 | 136L.00 | Alcohol intake within recommended sensible limits |
| 111933 | 8Cx0200 | Family wellbeing discussion about alcohol |
| 16587 | ZV11311 | [V]Problems related to lifestyle alcohol use |
| 37264 | 8CE1.00 | Alcohol leaflet given |
| 102564 | 8CAM000 | Advised to abstain from alcohol consumption |
| 11140 | ZG23100 | Advice on alcohol consumption |
| 967 | 1367 | Stopped drinking alcohol |
| 19489 | 6892 | Alcohol consumption screen |
| 97163 | ZC22200 | Advice to change alcoholic drink intake |
| 9264 | 68S..00 | Alcohol consumption screen |
| 64409 | Z191400 | Self-monitoring of alcohol intake |
| 96107 | 38D2.00 | Single alcohol screening questionnaire |
| 19401 | 136R.00 | Binge drinker |
| 1618 | 1365 | Heavy drinker - 7-9u/day |
| 99877 | 136b.00 | Feels should cut down drinking |
| 100493 | 38Df.00 | Five-shot questionnaire on heavy drinking |
| 8999 | 136P.00 | Heavy drinker |
| 12984 | 136Q.00 | Very heavy drinker |
| 12977 | 1366 | Very heavy drinker - >9u/day |
| 102665 | 136a.00 | Increasing risk drinking |
| 102448 | 136c.00 | Higher risk drinking |

**Code list for family planning and contraception**

| **medcode** | **readcode** | **Read term** |
| --- | --- | --- |
| 63892 | ZLE4.00 | Discharge from family planning service |
| 32726 | ZL15.00 | Under care of family planning doctor |
| 17836 | 8HTD.00 | Referral to family planning clinic |
| 22785 | ZL55.00 | Referral to family planning doctor |
| 17499 | ZL94.00 | Seen by family planning doctor |
| 22864 | ZLD2F00 | Discharge by family planning doctor |
| 8079 | 61...11 | Family planning |
| 9634 | 9N0U.00 | Seen in family planning clinic |
| 22948 | 61Z..00 | Contraception NOS |
| 11507 | 61B..11 | Depot contraception |
| 93451 | 8CEE.00 | Contraception leaflet given |
| 103009 | 679K500 | Education for withdrawal contraception |
| 96915 | 8CAw.00 | Advice about long acting reversible contraception |
| 4688 | 617..11 | Sheath contraception |
| 19501 | 614Z.00 | Oral contraception NOS |
| 93549 | 8CEF.00 | Intrauterine device contraception leaflet given |
| 39559 | 8CED.00 | Emergency contraception leaflet given |
| 5666 | 614..11 | Oral contraception |
| 103292 | 61S..00 | Contraception method not decided |
| 15806 | 61AZ.00 | Post-coital contraception NOS |
| 22947 | 61B5.00 | Depot contraception stopped |
| 1848 | 6777 | Contraception counselling |
| 2446 | 61J..00 | Contraception contraindicated |
| 12991 | 612..00 | Contraception not needed |
| 29033 | 61E..00 | Sympto-thermal contraception |
| 106646 | 8BV..00 | Emergency contraception indicated |
| 6477 | 614F.00 | Emergency contraception advice |
| 102309 | 8CAw100 | Verbal advice about long acting reversible contraception |
| 22935 | 61FZ.00 | Post-coital contraception NOS |
| 13005 | 616..12 | Diaphragm contraception |
| 6255 | 616..11 | CAP contraception |
| 22941 | 61C..11 | Spermicide alone contraception |
| 10614 | 615..11 | Coil contraception |
| 11810 | 961..11 | FP1001 - contraception claim |
| 20581 | 6146 | Oral contraception - problem |
| 29820 | SP03217 | Contraception IUCD causing bleeding |
| 54456 | 61P..00 | No current contraception |
| 102621 | 1P77.00 | Reason for no contraception |
| 102867 | 61V..00 | Problem with contraception |
| 17573 | 615..12 | IUD contraception |
| 6759 | 61A..00 | Post-coital contraception |
| 41 | 61...00 | Contraception |
| 95989 | 67P2.00 | Discussion about contraception injection |
| 102308 | 8CAw200 | Written advice about long acting reversible contraception |
| 110589 | 61c..00 | Barrier contraception method |
| 19497 | 618..00 | Rhythm method contraception |
| 102604 | 61X..00 | Planned contraception method |
| 13007 | 6145 | Oral contraception -no problem |
| 102367 | 61Y..00 | Uses contraception |
| 29417 | 61L..00 | Contraception status unknown |
| 108120 | 8T0F.00 | Referral to contraception and sexual health service |
| 8538 | 61M..00 | Emergency contraception |
| 13000 | 612Z.00 | Contraception not needed NOS |
| 19496 | 619..00 | Withdrawal contraception |
| 12998 | 61H..00 | Contraception: female sterilis |
| 105422 | 8CPA.00 | Discussion about risks of combined oral contraception |
| 110889 | 61d..00 | Natural contraception |
| 102368 | 61R..00 | Intrauterine system contraception |
| 96937 | 8CEG.00 | Long acting reversible contraception leaflet given |
| 6358 | 614..12 | Pill - oral contraception |
| 105778 | 14Og.00 | At risk of unwanted pregnancy |

**Code list for smear test/cytology appointments**

| **medcode** | **readcode** | **Read term** |
| --- | --- | --- |
| **3471** | **4K3..00** | **Cervical smear - inflam.change** |
| **10970** | **R150.00** | **[D]Nonspecific abnormal Papanicolaou cervical smear** |
| **19481** | **9O8f.00** | **Cervical smear screening verbal invitation** |
| **10484** | **4K36.12** | **HPV changes: cervical smear** |
| **12907** | **4K36.11** | **Herpes: cervical smear** |
| **7610** | **4K2..11** | **Dyskaryosis on cervical smear** |
| **41940** | **R150z00** | **[D]Nonspecific abnormal Papanicolaou cervical smear NOS** |
| **7003** | **9O89.11** | **SMEAR ABNORMAL - PT. NOTIFIED** |
| **9636** | **4K34.00** | **Cervical smear - candida** |
| **12932** | **4K2B.00** | **Cervical smear endocervical cells absent** |
| **4132** | **685..12** | **Cervical smear screen** |
| **8023** | **4K36.00** | **Cervical smear - wart virus** |
| **6211** | **ZV76212** | **[V]Routine cervical smear** |
| **42419** | **ZV76211** | **[V]Routine Papanicolaou smear** |
| **33256** | **4K48.00** | **Cx. smear: colposcopy needed** |
| **11835** | **7E2A300** | **Vaginal vault smear** |
| **12931** | **4K22.11** | **Smear NAD: no endocervic cells** |
| **12906** | **4K31.00** | **Cervical smear-no inflammation** |
| **12900** | **4K33.00** | **Cervical smear - trichomonas** |
| **107312** | **4K2R.00** | **Cervical smear - human papillomavirus positive** |
| **12914** | **4KA4.00** | **Vaginal vault smear abnormal** |
| **5190** | **4K23.00** | **Cerv.smear: mild dyskaryosis** |
| **12919** | **9O89.00** | **SMEAR ABNORMAL - PATIENT TOLD** |
| **107348** | **4K2P.00** | **Cervical smear - ?non-cervical type glandular neoplasia** |
| **102879** | **4KK..00** | **Vaginal vault smear action needed** |
| **104617** | **4K2F.00** | **Cervical smear pus cells present** |
| **26469** | **4K2D.00** | **Cervical smear transformation zone cells present** |
| **1088** | **4KA..00** | **Vaginal vault smear result** |
| **26468** | **4K2C.00** | **Smear NAD - no endocervical cells** |
| **3644** | **4K21.00** | **Cervical smear:inadequate spec** |
| **2599** | **7E2A200** | **Papanicolau smear NEC** |
| **12293** | **9O8S.00** | **Cervical smear defaulter** |
| **11** | **4K22.00** | **Cervical smear: negative** |
| **4288** | **4K26.00** | **Cervical smear: ? gland neopl.** |
| **15379** | **4K39.00** | **Cervical smear - gardnerella** |
| **107261** | **4K29000** | **Cervical smear - borderline change in squamous cells** |
| **107412** | **4K2Q.00** | **Cervical smear - human papillomavirus negative** |
| **12418** | **4K4Z.00** | **Cervical smear action NOS** |
| **17290** | **42b6.00** | **Percentage smear cells** |
| **55766** | **4K49.00** | **Cx. smear:cervical biopsy need** |
| **107295** | **4K2J.00** | **Cervical smear - low grade dyskaryosis** |
| **105903** | **4K2H.00** | **Cervical smear epithelial cells absent** |
| **271** | **7E2A211** | **Cervical smear NEC** |
| **12940** | **4K3A.00** | **Cervical smear: koilocytosis** |
| **12928** | **4K3C.00** | **Cervical smear - moderate inflammation** |
| **12934** | **4K2A.00** | **Cervical smear endocervical cells present** |
| **104665** | **4K2G.00** | **Cervical smear red blood cells present** |
| **32971** | **4K2E.00** | **Cervical smear transformation zone cells absent** |
| **73580** | **R150100** | **[D] Koilocytosis - cervical smear** |
| **22931** | **4K35.11** | **Viral changes on cerv. smear** |
| **22930** | **4KA3.00** | **Vaginal vault smear-atrophic** |
| **107344** | **4K29100** | **Cervical smear - borderline change in endocervical cells** |
| **12896** | **4K28.00** | **Cerv.smear: mod.dyskaryosis** |
| **28220** | **9O8R.00** | **Smear normal - pt. notified** |
| **8578** | **4K2..00** | **Cervical smear result** |
| **7728** | **4K24.00** | **Cerv.smear: severe dyskaryosis** |
| **17712** | **4K27.00** | **Cervical smear:atrophic change** |
| **5276** | **4K25.00** | **Cerv.smear:severe dysk.?inv.ca** |
| **109144** | **9EVC.00** | **Cervical smear report received** |
| **12904** | **4KAZ.00** | **Vaginal vault smear NOS** |
| **49408** | **9O8W.00** | **Cervical smear to continue post hysterectomy** |
| **12897** | **4K3Z.00** | **Cervical smear - inflam. NOS** |
| **66796** | **4K4A.00** | **Cx. smear: uterine curet. need** |
| **64151** | **R151.00** | **[D]Nonspecific abnormal Papanicolaou smear of other origin** |
| **107497** | **4K2N.00** | **Cervical smear - ?endocervical type glandular neoplasia** |
| **107363** | **4K2K.00** | **Cervical smear - high grade dyskaryosis (moderate)** |
| **98795** | **9O8i.00** | **Annual cervical smear required** |
| **12929** | **4K3B.00** | **Cervical smear - mild inflammation** |
| **35460** | **R150.12** | **[D]Papanicolaou smear - nonspecific abnormality** |
| **16820** | **R150.11** | **[D]Cervical smear - nonspecific abnormality** |
| **12912** | **4K38.00** | **Cervical smear - actinomyces** |
| **5446** | **R150000** | **[D]Dyskaryotic cervical smear** |
| **20403** | **4K2..12** | **Dysplasia (dysk.)on cerv.smear** |
| **12917** | **4K2Z.00** | **Cervical smear result NOS** |
| **12909** | **4KA2.00** | **Vaginal vault smear-inadequate** |
| **4823** | **4K29.00** | **Cerv.smear: borderline changes** |
| **46299** | **9O8D.00** | **Abn.smear, noted,recall delete** |
| **104378** | **9Ndx.00** | **Informed consent for cervical smear given** |
| **16030** | **7E2A000** | **Exam female genital tract under anaesth & Papanicolau smear** |
| **12908** | **4K32.00** | **Cervical smear-severe inflamm.** |
| **12911** | **4K35.00** | **Cerv.smear - viral infl.unsp.** |
| **12933** | **4K2Z.11** | **Nuclear abnormality on smear** |
| **12905** | **4KA1.00** | **Vaginal vault smear negative** |
| **12927** | **4K27.11** | **Atrophic change on cerv.smear** |
| **7308** | **4K4..00** | **Cervical smear - action needed** |
| **107264** | **4K2L.00** | **Cervical smear - high grade dyskaryosis (severe)** |
| **12898** | **685E.00** | **Cervical smear status unknown** |
| **41332** | **9O8V.00** | **Place cervical smear taken** |
| **26289** | **8CEA.00** | **Cervical smear information leaflet given** |
| **19483** | **4K37.00** | **Cervical smear - herpes** |
| **2173** | **6859.11** | **Cervical cytology examination** |
| **45789** | **4JRL.00** | **Cervical cytology screening test** |
| **12926** | **685..11** | **Cervical cytology screen** |
| **36920** | **4149** | **Cervical cytology sample sent to laboratory** |
| **17905** | **ZG52100** | **Advice on cervical cytology** |
| **19142** | **4K55.00** | **Cervical cytology test** |
| **42991** | **685R.00** | **Liquid based cervical cytology screening** |
| **7355** | **6793** | **Health ed. - cervical cytology** |
| **55061** | **4JRK.00** | **Gynaecology cytology screening test** |
| **60442** | **9O8..12** | **Cytology-cervical-admin** |
| **12921** | **6854** | **Ca cervix screen - wanted** |
