# Supplementary material for: Pre-pregnancy care in general practice in England: cross-sectional observational study using administrative routine health data
Source: BMC Public Health. 2025 Mar 22;25:1101. doi: 10.1186/s12889-025-21728-1 (PMC11929985; doi:10.1186/s12889-025-21728-1)
Supplement: Supplementary file 3 — Additional file 3. Code lists for active management of medical conditions. [file 12889_2025_21728_MOESM3_ESM.docx]

## Additional file 3 Code lists for active management of medical conditions

**Code list for active management of smoking**

enttype = 6 Health promotion - Smoking

enttype = 4 Smoking

enttype = 23 & data2 = "2" Advice given & Type of advice: Smoking

**Code list for active management of obesity**

enttype = 13 Weight

enttype = 476 Waist circumference

enttype = 9 Health promotion - Overweight

enttype = 23 & (data2 = "3" or data2 = "9") Advice given & Type of advice: Diet or Exercise

enttype = 29 Diet

enttype = 30 Exercise

**Code list for active management of diabetes**

enttype = 275 HbA1c - diabetic control

enttype = 22 Diabetes annual check

enttype = 26 Current Diabetes status

enttype = 65 Diabetic consultation

enttype = 91 Diabetes concerns

enttype = 97 Insulin dosage

enttype = 470 Diabetic retinopathy screening

enttype = 18 Diabetic register

**Code list for active management of hypertension**

enttype = 57 Angina

enttype = 64 CV / BP consultation

enttype = 15 Hypertension register

enttype = 1 Blood pressure

enttype = 130 Pre - treatment BP

enttype = 475 Target blood pressure

enttype = 8 Health promotion – Hypertension

**Code list for active management of asthma**

enttype = 25 Current Asthma status

enttype = 27 Asthma diagnosis

enttype = 38 Inhaler ability

enttype = 45 Night cough

enttype = 62 Asthma consultation

enttype = 113 Asthma last attack

enttype = 121 Asthma management at 30% peak flow

enttype = 122 Asthma management at 50% peak flow

enttype = 123 Asthma management at 80% peak flow Clinical Asthma

enttype = 138 Asthma concerns

enttype = 309 PF best ever

enttype = 310 PF predicted

enttype = 311 PF current

enttype = 463 Asthma Limiting Activity

enttype = 464 Asthma Night time symptoms

enttype = 465 Asthma Daytime symptoms

enttype = 477 Lung function post steriods

enttype = 486 Lung function pre-steroids

enttype = 19 Asthma register

**Code list for active management of epilepsy**

enttype = 205 Valproate

enttype = 140 Epilepsy register

enttype = 28 Epilepsy managed by

enttype = 86 Fit details

enttype = 94 Last fit
