# Supplementary material for: Pre-pregnancy care in general practice in England: cross-sectional observational study using administrative routine health data
Source: BMC Public Health. 2025 Mar 22;25:1101. doi: 10.1186/s12889-025-21728-1 (PMC11929985; doi:10.1186/s12889-025-21728-1)
Supplement: Supplementary file 4 — Additional file 4. Supplementary Table Provision of pre-pregnancy care by characteristics of women in those who became pregnant in 2017–2018. [file 12889_2025_21728_MOESM4_ESM.docx]

| **Additional file 4 Supplementary Table Provision of pre-pregnancy care by characteristics of women in those who became pregnant in 2017-2018** | | | | |
| --- | --- | --- | --- | --- |
|  | **Specific pre-pregnancy care and advice received** | | |  |
|  | **No** |  | **Yes** |  |
|  | **Number** | **%** | **Number** | **%** |
| **Age** |  |  |  |  |
| <20 yrs | 314 | 96.9 | 10 | 3.1 |
| 20-24 yrs | 2023 | 96.3 | 77 | 3.7 |
| 25-29 yrs | 3494 | 93.6 | 239 | 6.4 |
| 30-34 yrs | 4077 | 91.0 | 405 | 9.0 |
| 35-39 yrs | 2529 | 90.1 | 279 | 9.9 |
| 40-44 yrs | 695 | 89.7 | 80 | 10.3 |
| >=45 yrs | x | >95.0 | x | <5.0 |
| **Ethnic group** |  |  |  |  |
| White British | 6676 | 92.3 | 559 | 7.7 |
| White other | 1252 | 91.3 | 120 | 8.8 |
| Mixed | 177 | 94.7 | 10 | 5.4 |
| Asian or Asian british | 769 | 87.7 | 108 | 12.3 |
| Black or Black british | 479 | 93.2 | 35 | 6.8 |
| Chinese or other | 235 | 92.2 | 20 | 7.8 |
| *Missing* | 3645 | 93.8 | 241 | 6.2 |
| **Practice IMD quintiles** |  |  |  |  |
| 1 (least deprived) | 2098 | 88.6 | 269 | 11.4 |
| 2 | 2192 | 90.7 | 226 | 9.4 |
| 3 | 2574 | 92.7 | 202 | 7.3 |
| 4 | 2374 | 94.4 | 141 | 5.6 |
| 5 | 3995 | 94.0 | 255 | 6.0 |
| **BMI (kg/m^2^), mean (SD)** |  |  |  |  |
| <18.5 | 523 | 94.2 | 32 | 5.8 |
| 18.5-24.9 | 5813 | 91.9 | 516 | 8.2 |
| 25-29.9 | 3024 | 92.1 | 261 | 8.0 |
| ≥30 | 2565 | 92.4 | 211 | 7.6 |
| *Missing* | 1308 | 94.7 | 73 | 5.3 |
| **Smoking** |  |  |  |  |
| Never smokers | 6810 | 91.5 | 636 | 8.5 |
| Former smokers | 3303 | 91.6 | 305 | 8.5 |
| Current smokers | 2919 | 95.3 | 143 | 4.7 |
| *Missing* | x | >95.0 | x | <5.0 |
| **Number of previous pregnancies** |  |  |  |  |
| 0 | 3588 | 88.6 | 461 | 11.4 |
| 1 or more | 9645 | 93.9 | 632 | 6.2 |

Note: numbers have been replaced with ’x’ to suppress small values (<10) and prevent deduction of these values from larger totals
